# Quality Assessment of an Immunotherapy Toxicity Clinic: A Clinical Audit at Castle Hill Hospital Evaluating Adherence to Clinical Guidelines, Documentation Standards, and Clinical Outcomes

**DOI:** 10.7759/cureus.114844

**Published:** 2026-08-20

**Authors:** Ahmed Hussein

**Affiliations:** 1 Queen's Centre for Oncology and Haematology, Castle Hill Hospital, Hull University Teaching Hospitals, Hull, GBR

**Keywords:** cancer immunotherapy, quality assessment, retrospective audit, toxicity clinic, treatment related toxicity

## Abstract

Immunotherapy has developed as a cancer treatment by enhancing the body's immune system to attack cancer cells. Checkpoint inhibitors (e.g., programmed cell death protein-1 (PD-1), programmed death-ligand 1 (PD-L1), cytotoxic T-lymphocyte antigen-4 (CTLA-4)) have shown very good outcomes in treating several tumour types. However, their use can cause a unique category of toxicities, immune-related adverse events (irAEs). These differ from chemotherapy-related side effects, may affect any organ, and require specialised recognition and management.

This audit reviews the performance of a dedicated Immunotherapy Toxicity Clinic at Queen’s Centre for Oncology and Haematology, Hull University Teaching Hospitals. It includes all oncology patients referred between January 1 and June 30, 2024. Using a validated clinical tool, we assess the types, severity, and outcomes of irAEs, as well as adherence to protocols and quality of documentation.

This work highlights current practice, identifies gaps, and provides a quality improvement tool for managing patients on immunotherapy.

## Introduction

Cancer immunotherapy has fundamentally transformed oncological practice over the past two decades, offering durable responses in tumour types that were previously refractory to conventional treatment [1].

Among the various immunotherapeutic strategies developed, immune checkpoint inhibitors (ICIs) have emerged as the most widely adopted, with established efficacy across a broad range of malignancies including melanoma, non-small cell lung cancer (NSCLC), renal cell carcinoma, and urothelial carcinoma [2-4].

ICIs function by blocking inhibitory receptors on T cells, most notably cytotoxic T-lymphocyte antigen-4 (CTLA-4) and programmed cell death protein-1 (PD-1) or its ligand programmed death-ligand 1 (PD-L1), thereby restoring and amplifying anti-tumour immune responses [5]. Ipilimumab, the first approved CTLA-4 inhibitor, demonstrated a landmark survival benefit in metastatic melanoma in 2011 [2].

Since then, PD-1 inhibitors including pembrolizumab and nivolumab, as well as PD-L1 inhibitors such as atezolizumab and durvalumab, have received regulatory approval across an expanding list of indications, both as monotherapy and in combination regimens [6,7].

However, the mechanism by which ICIs enhance immune activity is inherently non-selective. By disrupting the regulatory checkpoints that maintain peripheral immune tolerance, these agents predispose patients to a distinct category of treatment-related side effects known as immune-related adverse events (irAEs) [8].

These toxicities differ fundamentally from those associated with conventional chemotherapy; they are immune-mediated, can affect virtually any organ system, and may arise at any point during or after treatment [9].

Commonly affected systems include the skin, gastrointestinal tract, liver, endocrine glands, and lungs, though rarer manifestations involving the heart, kidneys, nervous system, and haematopoietic system have also been well documented [10].

The incidence and severity of irAEs vary considerably depending on the class of ICI, the dose used, and whether agents are administered in combination. Combination regimens targeting both CTLA-4 and PD-1 carry the highest toxicity burden, with rates of significant adverse events exceeding those seen with monotherapy [11].

Grade 3 or 4 irAEs, which may necessitate treatment discontinuation, hospitalisation, and high-dose immunosuppression, occur in approximately 20-60% of patients receiving combination therapy [6].

Mortality directly attributable to irAEs, although uncommon, has been reported particularly in the context of immune-mediated myocarditis and severe pneumonitis [12,13].

Effective management of irAEs requires prompt recognition, accurate grading using the Common Terminology Criteria for Adverse Events (CTCAE), and timely implementation of evidence-based treatment protocols [14].

Corticosteroids remain the cornerstone of management for most moderate to severe irAEs, with additional immunosuppressive agents such as infliximab, mycophenolate mofetil, or intravenous immunoglobulin reserved for steroid-refractory cases [9].

Both the European Society for Medical Oncology (ESMO) and the UK Oncology Nursing Society (UKONS) have published detailed clinical guidelines to standardise the recognition and management of irAEs across oncology centres [9,15].

In recognition of the growing complexity and frequency of immunotherapy-related toxicity, a dedicated Immunotherapy Toxicity Clinic was established at Queen's Centre for Oncology and Haematology, Castle Hill Hospital, Hull University Teaching Hospitals NHS Trust in 2021. This clinic provides a structured follow-up service for patients experiencing irAEs, aiming to ensure consistent, guideline-driven care within a specialist setting.

Despite the availability of national and international guidelines, real-world adherence to irAE management protocols remains variable [7]. Gaps in documentation, incomplete investigation workup, delayed specialist referral, and inconsistent steroid monitoring have been identified as recurring quality concerns in the delivery of immunotherapy toxicity services [16]. Clinical audit represents an essential tool for evaluating the quality of care delivered against defined standards and identifying opportunities for systematic improvement [10].

This audit aimed to evaluate the performance and quality of service of the Immunotherapy Toxicity Clinic at Queen's Centre for Oncology and Haematology, Castle Hill Hospital, Hull University Teaching Hospitals, with respect to adherence to UKONS clinical guidelines, documentation quality, and clinical outcomes in patients referred with irAEs between January and June 2024.

## Materials and methods

Study design

This study was conducted as a retrospective clinical audit using a structured audit cycle framework. The audit was designed to measure current clinical practice against defined national standards, identify areas of underperformance, and generate an action plan to support service improvement. Ethical approval was not required as the work was classified as a clinical audit of routine practice rather than a research study. Institutional approval was obtained from Hull University Teaching Hospitals NHS Trust prior to data collection.

Study setting

The audit was conducted at the Immunotherapy Toxicity Clinic, Queen's Centre for Oncology and Haematology, Castle Hill Hospital, Hull University Teaching Hospitals NHS Trust. The clinic operates on a biweekly basis and is staffed primarily by a physician associate under the supervision of a senior oncology consultant. The clinic was established in 2021 with the specific remit of monitoring and managing patients experiencing irAEs following treatment with ICIs.

Audit standard

Given the existence of multiple international and national guidelines for the management of immunotherapy-related toxicities, the UKONS immunotherapy toxicity guidelines were selected as the primary audit standard [15]. UKONS guidelines were chosen as they represent the most applicable nationally recognised framework for oncology nursing and physician practice within the NHS setting. Adherence to the local institutional immunotherapy toxicity protocol, last updated in 2019, was assessed in parallel to allow direct comparison between national and local guideline compliance and to identify any gaps in the local protocol itself.

Patient population

All patients referred to and managed within the Immunotherapy Toxicity Clinic during the audit period of 1 January 2024 to 30 June 2024 were included. No exclusion criteria were applied, ensuring that the full case-mix encountered by the clinic was captured. Importantly, although patients were identified based on their attendance within this timeframe, the full clinical management journey for each patient was evaluated, including cases where the initial toxicity episode or management plan had commenced prior to 1 January 2024. This approach ensured that clinical decision-making was assessed comprehensively rather than as a cross-sectional snapshot of a single clinic visit.

Data collection

A structured data collection tool was developed prospectively for the purposes of this audit. Data were extracted from electronic patient records, clinic letters, and immunotherapy toxicity clinic documentation held within the hospital information system. Variables were collected across several domains as outlined below.

Patient demographics

The following demographic variables were recorded for each patient: age at time of clinic attendance, sex, primary cancer diagnosis, and cancer stage at the time of immunotherapy initiation.

Immunotherapy details

Data were collected on the specific ICI agent or combination regimen received, the intent of treatment (neoadjuvant, adjuvant, maintenance, or palliative), and whether any concurrent anticancer treatments such as chemotherapy or targeted therapy were being administered alongside immunotherapy.

Toxicity characteristics

The type and number of irAEs experienced by each patient were documented. Toxicities recorded included dermatitis, enterocolitis or diarrhoea, hypothyroidism, hyperthyroidism, adrenal insufficiency, hepatitis, pneumonitis, myocardial toxicity, myalgia, acute kidney injury, pancreatitis, and other less common toxicities including arthritis, arthralgia, polymyalgia rheumatica, retinopathy, and cytokine release syndrome. The grade of the presenting toxicity at the time of initial assessment was recorded using the CTCAE v5.0, ranging from Grade 1 (mild) to Grade 4 (life-threatening) [14]. The time from initiation of immunotherapy to onset of toxicity was calculated in weeks.

Site of initial assessment

The clinical setting in which each patient received their first formal assessment for the presenting irAE was recorded. Categories included the Acute Cancer Assessment Unit (ACAU), inpatient ward, primary oncology consultant clinic, day treatment unit, the immunotherapy toxicity clinic itself, or assessment by a systemic anticancer therapy clinical nurse specialist (SACT CNS).

Documentation quality

The completeness of clinical documentation within the immunotherapy toxicity clinic records was assessed for each patient. Documentation was considered complete when all core components were present within the clinic record. These components included patient identification details, primary cancer diagnosis, name and regimen of immunotherapy, type and grade of irAE, results of relevant investigations, details of treatment decisions including whether immunotherapy was held or discontinued, immunosuppressive treatment prescribed including drug, dose, and planned tapering schedule, evidence of symptom progress at follow-up, and whether specialist referral had been arranged. Records were classified as having incomplete documentation if any of these domains were absent or insufficiently detailed. The specific domain of missing information was also categorised, distinguishing between gaps in patient details and gaps in management progress documentation.

Protocol adherence

Adherence to the UKONS immunotherapy toxicity guidelines was assessed for each patient with a confirmed irAE and an identifiable management protocol. Cases were classified as adherent if the investigations performed and the treatment decisions made were consistent with the UKONS recommendations for the relevant toxicity type and grade. Cases were classified as non-adherent if one or more required elements of the protocol were absent or significantly delayed. The specific nature of each deviation was categorised as relating to investigations only, treatment only, or both investigations and treatment. The toxicity type associated with each deviation was also recorded to identify which irAE categories were most frequently associated with protocol non-compliance. Adherence to the local 2019 institutional protocol was assessed separately using the same methodology.

Management details

The following management variables were recorded for each patient: whether corticosteroids were prescribed and the total duration of steroid therapy in weeks, whether additional immunosuppressive agents were used and which agents were selected, whether immunotherapy was temporarily held and the duration of the hold in weeks, and whether immunotherapy was permanently discontinued. Whether specialist referral to another clinical discipline was made was also documented.

Clinical outcomes

The following outcome measures were assessed. The presence of refractory toxicity was defined as failure to respond adequately to first-line corticosteroid therapy, necessitating escalation to second-line immunosuppression or resulting in prolonged irAE duration. Mortality directly attributable to immunotherapy toxicity was recorded separately from mortality related to underlying cancer progression, in order to avoid overestimation of toxicity-related death and to maintain clinical accuracy of attribution [10]. The total number of immunotherapy toxicity clinic attendances per patient was also recorded as a measure of healthcare utilisation.

Statistical analysis

Data were entered and analysed using IBM SPSS Statistics (version 26; IBM Corp., Armonk, NY, USA). Descriptive statistics were used to summarise all variables. Continuous variables including age, time to toxicity, steroid duration, ICI hold duration, and number of clinic visits were summarised using mean, standard deviation, median, minimum, and maximum values. Categorical variables were summarised using frequencies and percentages. Where relevant, the distribution of protocol deviations across toxicity types was examined to determine whether specific irAE categories were disproportionately associated with guideline non-adherence, with the aim of directing future targeted educational and quality improvement interventions.

## Results

In a cohort of 70 patients evaluated at the clinic, the primary function was to monitor progress following the first management plan in 65 patients (92.9%), whereas five patients received their management plan directly at the immunotherapy clinic (7.1%) (Table 1).

**Table 1:** Action taken at the immunotherapy clinic

Action	Frequency	Percent
Initiation of management plan (1)	5	7.1
Follow-up (2)	65	92.9
Total	70	100.0

In the context of toxicity management, the treatment plan for 35 patients shown a 50% non-adherence to the protocol, with two situations lacking a particular protocol (2.9%), one patient experiencing no toxicity (patient was seen for short synacthen test which resulted as normal), and one patient not receiving immunotherapy (mistakenly a patient with everolimus-induced pneumonitis was referred to the clinic) (1.4% each). Out of 35 patients, 23 (65.7%) exhibited deviations from the protocol for investigations, while 10 patients (28.6%) had deviations in treatment. Two (5.8%) patients deviated from the guidelines concerning treatment and investigation. Protocol deviations were observed in patients with colitis (10 patients, 28.6%), pneumonitis (7 patients, 20%), cardiac issues (2 patients, 5.7%), hepatitis (2 patients, 5.7%), dermatitis (2 patients, 5.7%), and in the bone protection protocol in 12 patients (34.2%). Compliance with local protocol was seen in 45 cases (64.3%) (Tables 2-4).

**Table 2:** Adherence to UK Oncology Nursing Society (UKONS) protocol [15] ICI: immune checkpoint inhibitor

Category	Frequency	Percent
No toxicity	1	1.4
No ICI	1	1.4
No	35	50.0
NO PROTOCOL	2	2.9
Yes	31	44.3
Total	70	100.0

**Table 3:** Deviation from guidelines

Deviation type	Frequency	Percent
Investigations only (1)	23	65.7
Investigations + treatment (1,2)	2	5.7
Treatment only (2)	10	28.6
Total	35	100.0

**Table 4:** Adherence to local protocol ICI: immune checkpoint inhibitor

Category	Frequency	Percent
No toxicity	1	1.4
No ICI	1	1.4
No	21	30.0
NO PROTOCOL	2	2.9
Yes	45	64.3
Total	70	100.0

Steroids were administered to 59 patients (84.3%), with a mean duration of 10.14 weeks. In nine patients, alternative immunosuppressive medications were used (12.9%) (Tables 5-7).

**Table 5:** Steroid dose

Steroid use	Frequency	Percent
No	11	15.7
Yes	59	84.3
Total	70	100.0

**Table 6:** Duration of steroids

Statistic	Value ( weeks)
Mean	10.14±8.56
Median	7.00
Minimum	3
Maximum	48

**Table 7:** Other immunosuppressive drugs

Other immunosuppressive drugs	Frequency	Percent
No	61	87.1
Yes	9	12.9
Total	70	100.0

Immunotherapy was discontinued in 18 patients (25.7%), with a minimum treatment interruption of two weeks, a maximum of 35 weeks, and a mean duration of 9.94 weeks. Immunotherapy was permanently discontinued in 40 patients (57.1%) over the entire term of cancer care (Tables 8-10).

**Table 8:** Hold ICI ICI: immune checkpoint inhibitor

Hold ICI	Frequency	Percent
No	52	74.3
Yes	18	25.7
Total	70	100.0

**Table 9:** Hold duration in weeks

Statistic	Value
Mean	9.94±0.67
Median	8.00
Minimum	2
Maximum	35

**Table 10:** Permanent ICI discontinuation ICI: immune checkpoint inhibitor

Permanently discontinued	Frequency	Percent
No	30	42.9
Yes	40	57.1
Total	70	100.0

Referrals to other specialties were made for 20 patients (28.6%), and refractory toxicity was seen in 27 patients (38.6%) at the time of data collection. Three individuals (4.3%) experienced fatalities directly attributable to immunotherapy toxicity (Tables 11-13).

**Table 11:** Referral to specialist

Referral to specialist	Frequency	Percent
No	50	71.4
Yes	20	28.6
Total	70	100.0

**Table 12:** Refractory adverse events

Refractory adverse events	Frequency	Percent
No	43	61.4
Yes	27	38.6
Total	70	100.0

**Table 13:** Mortality related to immunotherapy (IO) toxicity

Mortality related to IO toxicity	Frequency	Percent
Yes	3	4.3
No	67	95.7
Total	70	100.0

## Discussion

A detailed evaluation of the clinical outcomes and the toxicities associated with immunotherapy encountered by patients visiting the immunotherapy toxicity clinic at Queen's Centre for Oncology and Haematology (in Castle Hill Hospital) has been documented in the audit. It helped in understanding the consequences of the clinic's service in the supervision of patients on ICIs, the clinic's service in respect to the toxicity outcomes, clinic outcomes, protocol and documentation adherence, and overall clinic service. The clinic is primarily operated by a physician associate with the mentorship of a senior physician [10,15].

The disposition of cases (follow-up vs. initiating a new treatment plan) indicates most patients (93%) were seen for follow-up, which indicates that presently, the toxicity clinic is generating and operating primarily as a tracking and supportive service. This suggests that preliminary management actions are frequently performed outside of the clinic (i.e. ACAU, consultancy clinics, or among inpatients). Broaden the clinic's parameters to include more proactive measures-like collecting outstanding investigations along with quick referral to designated specialists-to enhance the overall system of managing irAE and streamline care according to the protocols [7,14].

As such, the focus on adherence to treatment protocols in this audit reflects the wider centre's irAE practice, rather than the clinic in isolation. Approximately 50% of patients deviated from UKONS management protocols, predominantly due to gaps in initial investigations and work-up (65.7% of deviations) rather than decisions around treatment. This suggests that the main quality gap is missing the timely completeness of early diagnostics that is vital for risk stratification and safe escalation to the use of immunosuppressive agents [7,15].

Most gaps in protocols were in colitis and pneumonitis, especially in the initial work-up. Examples were incomplete infectious screening in colitis, lack of timely endoscopy where indicated, and omitting TB screening in patients where infliximab might be needed. In pneumonitis, gaps included lack of screening for atypical infections and failure to obtain high-resolution CT scans. Comparable investigation gaps were also seen in myocarditis and hepatitis, with a few cases of the absence of adequate specialist referral pathways, which are well recognised to be a cornerstone in the management of high-risk irAEs [17-20].

One gap more directly linked to the toxicity clinic's follow-up role is in the monitoring and prevention of steroid-related adverse effects, particularly infection, osteoporosis, and hyperglycaemia. Reviewing other aspects of the patients' follow-up also indicated that the practice stayed within the boundaries of the current recommendations that examine the need for the prophylactic use of antibiotics after prolonged courses of steroids, as well as the need for tight control of diabetes. Nonetheless, for 12 patients, the absence of, or incomplete, monitoring and intervention (including incomplete or absent prescriptions, and/or absence of the appropriate investigations, such as DEXA scanning) represented one of the clear and actionable safety gaps within the follow-up of patients who are chronically immunosuppressed [21,22].

However, adherence to local guidance was higher than adherence to UKONS protocols (45 vs 35 patients). One possible explanation is that the most recent local guideline (2019) may be less prescriptive, may have fewer required elements to "pass" adherence, and may be less detailed and less up-to-date. Most importantly, it did not provide guidance on prolonged steroid monitoring, which may lead to variability on the follow-up safety measures and under-recognition of the risks associated with the steroids [10,15].

The assessment of the quality of the documentation was subjective, first via assessment of whether the clinical records captured the core components in clinical documentation including patient identification, diagnosis, type of immunotherapy, type and grade of toxicity, tests, treatment, and details of the symptoms, and details of the immunosuppressive medication, including duration and tapering plan, and whether progress was made in symptoms. Only 39 patients (55.7%) had complete documentation, which is less than expected. Case records for 17 patients did not have some fundamental patient details (e.g., type of cancer or type of immunotherapy) or complete details on management steps (e.g., steroid duration/tapering, whether a specialty referral was made) which can compromise the continuity of care, result in unsafe, poor decision-making, and poor auditability of the processes [7,15].

Documentation of referral to other specialties was only for 20 patients. This is lower than expected considering the multisystem nature of irAEs and the common necessity for integrated/combined management with gastroenterology, respiratory, cardiology, endocrinology, and hepatology. Understanding barriers to timely referral (process, access, knowledge, or documentation issues) could significantly enhance patient pathways and lessen the lag in conclusive investigations and treatment escalation [7,16].

Documentation lapse and deviation from the protocol require improvement in healthcare quality. Document focus guideline training, clinic role enhancement where all fundamental management like symptom control and steroid prescribing, practical documentation aids, and scheduled multidisciplinary reviews to standardize investigations, referrals, and follow-up safety monitoring. Updating the local immunotherapy toxicity protocol will help junior staff, nursing, and physician associates deliver evidence-based care efficiently [7,14,15].

Limitation of the study

This study has several limitations that should be considered when interpreting its findings. First, it was conducted as a single-centre retrospective audit at a tertiary oncology centre, which may limit the generalisability of the results to other institutions with different patient populations, staffing models, or clinical pathways. Second, the study relied on routinely collected electronic medical records; therefore, the completeness and accuracy of the data were dependent on the quality of clinical documentation, and some variables may have been incompletely recorded or subject to documentation bias.

In addition, the retrospective design prevented control over data collection and did not allow assessment of causal relationships between clinic performance and patient outcomes. The audit also covered a relatively limited study period, which may not fully reflect long-term trends in service performance or the impact of subsequent changes in clinical practice. Furthermore, patient-reported outcomes, quality-of-life measures, and patient satisfaction were not routinely available and therefore could not be evaluated. Despite these limitations, the audit provides a valuable assessment of real-world clinical practice, identifies areas for service improvement, and establishes a baseline for future re-audit following implementation of recommended changes.

## Conclusions

This audit demonstrated that the Immunotherapy Toxicity Clinic at Queen's Centre for Oncology and Haematology provides a functional and clinically important service for monitoring and supporting patients experiencing irAEs. However, significant opportunities for improvement were identified. Protocol adherence against UKONS guidelines was achieved in fewer than half of cases, with investigation-driven gaps representing the most frequent source of deviation, particularly in colitis and pneumonitis. Documentation was incomplete in nearly half of all patient records, and specialist referral rates remained lower than expected given the multisystem nature of irAEs. The local institutional protocol, last updated in 2019, requires revision to reflect current evidence and to incorporate guidance on steroid monitoring and bone protection. To address these findings, targeted interventions are recommended including structured documentation tools, mandatory guideline-based training for all clinic staff, a clearly defined specialist referral pathway, and an updated local protocol aligned with current UKONS standards. Following implementation of these improvements, a re-audit is planned to complete the audit cycle and evaluate the impact of these changes on the quality and safety of immunotherapy toxicity care delivery.
